# Preferences for different features of ENDS products by tobacco product use: a latent class analysis

**DOI:** 10.1186/s13011-022-00448-4

**Published:** 2022-03-09

**Authors:** Chineme Enyioha, Marcella H. Boynton, Leah M. Ranney, M. Justin Byron, Adam O. Goldstein, Christine E. Kistler

**Affiliations:** 1 Department of Family Medicine, University of North Carolina, 590 Manning Drive, University of North Carolina at Chapel Hill, Chapel Hill, NC 27599 USA; 2 Lineberger Comprehensive Cancer Center, University of North Carolina at Chapel Hill, Chapel Hill, NC USA; 3 Division of General Medicine and Clinical Epidemiology, Department of Medicine, UNC School of Medicine, University of North Carolina at Chapel Hill, Chapel Hill, North Carolina USA; 4 North Carolina Translational and Clinical Sciences Institute, University of North Carolina at Chapel Hill, Chapel Hill, North Carolina USA; 5 Department of Health Behavior, Gillings School of Global Public Health, University of North Carolina, Chapel Hill, NC USA

**Keywords:** ENDS, Tobacco products, Regulations, Cessation

## Abstract

**Background:**

From a public health perspective, electronic nicotine delivery devices (ENDS) use may be beneficial for some populations (e.g., smokers who fully switch to ENDS) but detrimental for others (e.g., nonsmokers). Understanding the importance placed on different ENDS product features by user groups can guide interventions and regulations.

**Methods:**

Participants were US adults who had used ENDS at least once and from a convenience sample drawn from a market research software in 2016. Participants chose between 9 different ENDS product features (harms of use, general effects of use, use as a cessation aid, initial purchase price, monthly cost, nicotine content, flavor availability, device design, and modifiability). A latent class analysis (LCA) identified subgroups of feature preferences and examined differences between groups by socio-demographics and tobacco product use.

**Results:**

Of the 636 participants, 81% were White, the median age was 42, and 65% were current cigarette smokers. The LCA identified a 4-class solution as the most appropriate model: (1) people with high nicotine dependence who preferred ENDS similar to combustible cigarettes, (2) people with moderate tobacco use who were interested in low nicotine ENDS (3) people who use ENDS and combustible tobacco who preferred lower price and flavored ENDS products, and (4) people who used ENDS predominantly, without a strong preference for any of the features presented.

**Conclusions:**

Tobacco use classes were associated with differences in preferences for ENDS features. These findings can inform regulations to reduce ENDS use among specific groups of people who use ENDS products.

## Introduction

Since the introduction of electronic nicotine delivery systems (ENDS) to the U.S. market in 2007 [1], use has risen dramatically [2, 3]. Despite suggestion of harms of ENDS use [4–7], ENDS use continues to be popular. ENDS are not harmless. Yet for many smokers who are unable or uninterested in quitting cigarettes, ENDS are regarded as a cessation aid or as a potentially less harmful alternative. Different groups of people may use ENDS for different reasons besides cessation such as lower cost compared to other tobacco products, stress reduction, curiosity, consideration for others, and convenience [8–11]. Other features such as flavors, nicotine content, device design, and functional similarities to cigarettes may also affect the appeal of ENDS to some people who use these products [12, 13]. Studies have also shown that some people who use ENDS stop using these products for several reasons including lack of interest in continuing to experiment with ENDS products, use of ENDS not the same feel as smoking cigarettes, inability to control dose of nicotine, technical concerns, side effects, unpleasant taste and cost [8, 14, 15]. Other less common reasons for discontinuing ENDS use include the persistent craving for tobacco cigarettes even while using ENDS, inability to quit or reduce smoking despite using ENDS while some others stop using ENDS because they quit smoking tobacco cigarettes [8]. One study investigated reason for discontinuation based on reasons for trying with a survey and found that those who started using ENDS for the purposes of quitting or cutting back on tobacco cigarettes were less likely to discontinue use compared with those whose reason for starting was based on curiosity or influence of others [8].

In 2016, the U.S. Food and Drug Administration (FDA) published a final “deeming” regulation that asserted authority to regulate ENDS and additional tobacco products [16]. While FDA cannot change tax levels, states and localities can use taxes and other tools to deter use [17]. Importantly, FDA set a deadline for all products that were not in the market as of February 15, 2007, which includes ENDS products, to submit “premarket tobacco product applications” for all new and existing products they wish to sell [18]. With the FDA’s ongoing consideration of product standards and the potential for regulations that aim to affect ENDS use among specific populations of people, it is important to understand how preferences for different features of ENDS differ by various groups of people who use ENDS. Understanding these differences may guide policymakers as they create regulations for ENDS products.

A method well-suited for classifying individuals into groups based on a set of personal characteristics is mixture modeling. Mixture models are a group of latent variable modeling techniques that allow a researcher to build typologies based on observed variables [19]. One subtype of mixture modeling, latent class analysis (LCA), is used to identify a set of discrete, non-overlapping groups (i.e. latent classes) based on a specific combination of individual characteristics. Individuals are assigned a class value based on the likelihood that their unique characteristics are best captured by their assigned class [20, 21].

The current study applies LCA to data collected in 2016 as part of a discrete choice experiment (DCE) of ENDS products using a convenience sample of people who use ENDS in the U.S. Data on stated preferences for features of ENDS products were used to construct the LCA classes, and sociodemographic factors, ENDS and other tobacco product use were used to further characterize class membership. This approach allowed us to identify product features especially attractive to different groups of people who use ENDS.

## Methods

### Study design

In a cognitively tested DCE embedded in a survey, participants indicated the importance of different ENDS features for their use [11, 12]. The survey was administered in August 2016 using Sawtooth software (Provo, Utah). Sawtooth is an online software platform to create and field discrete choice questions as well as assist with analysis of choice experiments. (Sawtooth) The experiment was designed from a best-worst scaling, case 2 DCE [22] The experiment was conducted with a total of 19 hypothetical products, with each product made up of 5 different features chosen from a total of 9 possible features. Specifically, the 9 features were: harms of use, general effects of use, use as a cessation aid, initial purchase price, monthly cost, nicotine content, flavor availability, device design, and modifiability [12]. Each respondent was presented with one product at a time, and for each product, was asked the following question “Please imagine this is a new e-cigarette that has just become available for purchase. When you look at the 5 features of the e-cigarette, which feature makes you most want to use the e-cigarette and which feature makes you least want to use the e-cigarette?” Each feature had 3 to 4 different levels relating to that feature. For example, the feature “one time purchase cost,” with 4 levels: $5, $55, $115, or $175 (Table 1).

**Table 1:** List of discrete choice experiment features and levels

Features	Levels	Theme
Harms of use	Less harmful on my body as compared to tobacco. Unknown harm to my body compared to tobacco. Same amount of harm on my body as compared to tobacco. More harmful on my body as compared to tobacco.	Health effect
General effects of use	Helps me breathe easier and my clothes do not smell like tobacco. Helps me breathe easier but my clothes smell like tobacco. Does not help me breathe easier but my clothes do not smell like tobacco. Does not help me breathe easier and still makes my clothes smell like tobacco.	
Tobacco cessation aid	7 of 10 people are able to quit tobacco cigarettes. 5 of 10 people are able to quit tobacco cigarettes. 2 of 10 people are able to quit tobacco cigarettes. People are not able to quit smoking tobacco cigarettes.	
Purchase price of product	$5 $55 $115 $175	Cost of use
Monthly cost of use	$5 $25 $65 $100	
Nicotine content	No nicotine (0 mg/ml0 Low nicotine (6 mg/ml) Medium nicotine (12 mg/ml) High nicotine (24 mg/ml)	Device features
Flavor availability	Available only without any flavoring Available in tobacco and menthol flavors Available in tobacco, menthol, fruit, candy, and other flavors	
Device design	Very similar in size, weight, and appearance and … Somewhat similar in size, weight, and appearance and …. Not similar at all in size, weight, and appearance	
Modifiability	It cannot be modified. Various parts can be modified	

The development of the list of features and levels and further details of the study design can be found elsewhere [11, 12]. The Institutional Review Board of UNC Chapel Hill School of Medicine approved this study.

### Participants

Participants were recruited from the survey panel of Research Now, an online research survey company. To be eligible for the study, participants had to be 18 years or older, reside in the United States, be able to speak and read English, have access to the internet, and report having used ENDS at least once in their lifetime.

### Survey measures

The survey included questions about participant sociodemographic factors including gender, age, race/ethnicity, education, household income, and general health. The survey also asked about tobacco product use including past and current ENDS use, the use of flavored ENDS, anticipated future ENDS use, use of other tobacco products, current cigarette smoking (cigarettes per day), age first started smoking, dual use of ENDS and combustible products, nicotine dependence, and past attempts to quit [23, 24].

### Analyses

The Choice Based Conjoint Hierarchical Bayesian module in Sawtooth Software typically uses a mixed effects multivariate, multinomial logit modeling approach to generate population mean “utilities,” which in this specific case represents the various ENDS features and their levels. In the current analysis we also used a mixed modeling approach; however, we used LCA to identify groups or “classes” of individuals based on their choices of the hypothetical ENDS products. Such an approach allowed us to explore whether different groups value certain constellations of ENDS features more than others. We iteratively tested 2 through 8 class LCA models and, using a combination of relative model fit statistics (e.g., Bayesian Information Criterion; BIC) and theory, identified the optimal class solution. We then compared the classes on key tobacco use and sociodemographic variables. Tobacco use classification was based on the heaviness of smoking index score, determined by the number of tobacco cigarettes smoked in a day and the time to first cigarette use after wake [23].

## Results

### Participant characteristics

A total of 636 met the inclusion criteria, and thus comprise the analytic sample for this study. The mean age was 42 years (SD ± 19) and 49% were female. Participants were majority White (81%) and 13.8% identified as Latino/Hispanic. Approximately one third of the sample had a college degree and 60% reported a household income under $60,000 per year (Table 2). Most participants (92%) reported that they had tried cigarette smoking and 65% were current smokers. In the sample, 63% reported ENDS use in the past 30 days. Of those who previously tried cigarettes (*n* = 588), 67% first started smoking before age 18 and 40% had used both ENDS and a combustible tobacco product in the past 30 days (dual use). Of the current cigarette smokers (*n* = 415), 59% reported smoking within 30 min of waking and 46% tried to quit tobacco cigarettes within the past 12 months (Table 3).

**Table 2:** Sample demographic characteristics – 4 class solution

	Total *N* = 636	Class 1 *n* = 169	Class 2 *n* = 159	Class 3 *n* = 147	Class 4 *n* = 161	*p* value
	n (%) or M ± SD	n (%) or M ± SD	n (%) or M ± SD	n (%) or M ± SD	n (%) or M ± SD	
Gender		3, 4	3	1	1	
Male	325 (51.1)	101 (59.8)	95 (59.7)	70 (47.6)	59 (36.6)	
Female	311 (48.9)	68 (40.2)	64 (40.3)	77 (52.4)	102 (63.4)	
Age		4	4	4	1, 2, 3	
in years	42.2 ± 19.4	45.5 ± 19.64	44.6 ± 20.34	46.2 ± 20.04	32.6 ± 13.6	
Age category		4	.	4	1, 3	
18–24	159 (25.0)	32 (18.9)	44 (27.7)	30 (20.4)	53 (32.9)	
25–34	150 (23.6)	37 (21.9)	25 (15.7)	29 (19.7)	59 (36.6)	
35–44	59 (9.3)	19 (11.2)	10 (6.3)	10 (6.8)	20 (12.4)	
45–54	46 (7.2)	9 (5.3)	15 (9.4)	13 (8.8)	9 (5.6)	
55–64	57 (9.0)	21 (12.4)	12 (7.5)	15 (10.2)	9 (5.6)	
65+	165 (25.9)	51 (30.2)	53 (33.3)	50 (34.0)	11 (6.8)	
Race		2, 4	1	4	1, 3	
White	515 (81.0)	156 (92.3)	123 (77.4)	121 (82.3)	115 (71.4)	
Black or African American	41 (6.4)	5 (3.0)	15 (9.4)	8 (5.4)	13 (8.1)	
Asian	38 (6.0)	3 (1.8)	8 (5.0)	5 (3.4)	22 (13.7)	
Other racial group	42 (6.6)	5 (3.0)	13 (8.2)	13 (8.8)	11 (6.8)	
Hispanic/Latino		4	.	4	1, 3	<.001
No	548 (86.2)	158 (93.5)	139 (87.4)	131 (89.1)	120 (74.5)	
Yes	88 (13.8)	11 (6.5)	20 (12.6)	16 (10.9)	41 (25.5)	
Education		.	.	.	.	.14
0 = < High school (HS) … 4 = Master’s degree	2.19 ± 0.99	2.27 ± 0.90	2.14 ± 0.95	2.05 ± 0.99	2.27 ± 1.09	
Education category		.	.	.	.	.16
< High school (HS)	16 (2.5)	2 (1.2)	3 (1.9)	5 (3.4)	6 (3.7)	
HS diploma or GED	148 (23.3)	30 (17.8)	39 (24.5)	42 (28.6)	37 (23.0)	
Associate degree or Some college	234 (36.8)	72 (42.6)	62 (39.0)	51 (34.7)	49 (30.4)	
Bachelor’s degree	175 (27.5)	50 (29.6)	42 (26.4)	38 (25.9)	45 (28.0)	
Master’s degree	63 (9.9)	15 (8.9)	13 (8.2)	11 (7.5)	24 (14.9)	
Household income	3.12 ± 1.85	3.19 ± 1.87	3.09 ± 1.82	2.91 ± 1.88	3.25 ± 1.83	
$0 to $29,999 per year	142 (22.3)	35 (20.7)	34 (21.4)	39 (26.5)	34 (21.1)	
$30,000 to $59,999 per year	239 (37.6)	65 (38.4)	65 (40.9)	52 (35.4)	57 (35.4)	
$60,000 or more per year	255 (40.1)	69 (40.9)	60 (37.7)	56 (38.1)	70(43.5)	
General health		4	4	4	1, 2, 3	<.001
0 = Poor … 4 = Excellent	2.70 ± 0.96	2.53 ± 0.974	2.60 ± 0.954	2.62 ± 0.944	3.04 ± 0.92	

Note: Bolded numbers indicate significant difference between class (e.g., 1 indicates difference *p* < .05 compared with Class 1)

**Table 3:** Tobacco use by 4 class membership

	TOTAL	CLASS 1	CLASS 2	CLASS 3	CLASS 4
	n (%) or M ± SD	n (%) or M ± SD	n (%) or M ± SD	n (%) or M ± SD	n (%) or M ± SD
ENDS PRODUCT USE	*N* = 636	*n* = 169	*n* = 159	*n* = 147	*n* = 161
ENDS use, past 30 days		**3, 4**		**1, 2**	**1**
No	235 (36.9)	80 (47.3)	69 (43.4)	45 (30.6)	41 (25.5)
Yes	401 (63.1)	89 (52.7)	90 (56.6)	102 (69.4)	120 (74.5)
ENDS use, past 30 days with flavor(s)		**2**	**1**	.	.
No flavor use	117 (18.4)	24 (14.2)	37 (23.3)	32 (21.8)	24 (14.9)
Flavor use	284 (44.7)	65 (38.5)	53 (33.3)	70 (47.6)	96 (59.6)
No ENDS use	235 (36.9)	80 (47.3)	69 (43.4)	45 (30.6)	41 (25.5)
Thinks will use ENDS in the next year		.	**3**	**2**	.
Definitely or probably not	174 (27.4)	46 (27.2)	59 (37.1)	34 (23.1)	35 (21.7)
Definitely or probably yes	462 (72.6)	123 (72.8)	100 (62.9)	113 (76.9)	126 (78.3)
Ever tobacco product use		**4**	.	.	**1**
Has never tried any tobacco products other than ENDS	48 (7.5)	7 (4.1)	8 (5.0)	12 (8.2)	21 (13.0)
Has tried at least one tobacco product other than ENDS	588 (92.5)	162 (95.9)	151 (95.0)	135 (91.8)	140 (87.0)
Tobacco cigarettes smoked per day		**2, 4**	**1, 3**	**2, 4**	**1, 3**
0 cigarettes (former or never smoker)	221 (34.7)	50 (29.6)	56 (35.2)	41 (27.9)	74 (46.0)
20 or fewer cigarettes per day	364 (57.3)	102 (60.3)	98 (61.6)	85 (57.9)	79 (49.1)
21 or more cigarettes per day	51 (8.0)	17 (10.1)	5 (3.1)	21 (14.3)	8 (5.0)
TOBACCO PRODUCT USE, *people with a history of use*	*N* = 588	*n* = 162	*n* = 151	*n* = 135	*n* = 140
Age first started smoking		**4**	.	.	**1**
< 14	58 (9.9)	14 (8.6)	20 (13.2)	12 (8.9)	12 (8.6)
14–15	140 (22.0)	53 (32.7)	29 (19.2)	41 (30.4)	17 (12.1)
16–17 years	223 (35.1)	57 (35.2)	62 (41.1)	57 (42.2)	47 (33.6)
18–24 years	125 (19.7)	31 (19.1)	35 (23.2)	17 (12.6)	58 (30.0)
25 years or older	42 (7.1)	7 (4.3)	5 (3.3)	8 (5.9)	22 (15.7)
Used both ENDS and at least one tobacco cigarette in the past 30 days		**3**	**3**	**1, 2**	
No	334 (56.8)	100 (59.2)	93 (61.6)	62 (45.9)	79 (56.4)
Yes	254 (39.9)	62 (36.7)	58 (38.4)	73 (54.1)	61 (43.6)
CIGARETTE USE, *current smokers*	*N* = 415	*n* = 119	*n* = 103	*n* = 106	*n* = 87
Number of minutes to first cigarettes		**2**	**1, 3**	**2**	.
< 5 min	64 (15.4)	20 (16.8)	11 (10.7)	20 (18.9)	13 (14.9)
6–30 min	180 (43.4)	57 (47.9)	39 (37.9)	46 (43.4)	38 (43.7)
31–60 min	62 (14.9)	17 (14.3)	10 (9.7)	16 (15.1)	19 (21.8)
> 60 min	109 (26.3)	25 (21.0)	43 (41.7)	24 (22.6)	17 (19.5)
Ever tried to quit smoking in the past		**2, 3, 4**	**1**	**1**	**1**
Yes, was successful	152 (36.6)	35 (29.4)	42 (40.8)	35 (33.0)	40 (46.0)
Yes, was not successful	203 (48.9)	74 (62.2)	47 (45.6)	50 (47.2)	32 (36.8)
No	60 (14.5)	10 (8.4)	14 (13.6)	21 (19.8)	15 (17.2)

Note: Bolded numbers indicate significant difference between class (e.g., 1 indicates difference *p* < .05 compared with Class 1)

### LCA findings

**Fig. 1:**
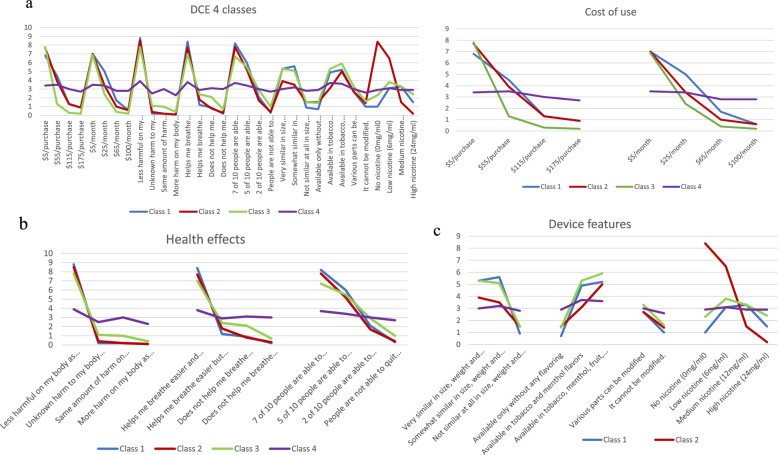
4 class solution for all features. **a**: 4 class solution for features related to cost of use. **b**: 4 class solution for features related to health effects. **c**: 4 class solution for features related to the device. Class 1: People with high nicotine dependence. Class 2: People with moderate tobacco use. Class 3: People who use ENDS and combustible tobacco. Class 4: People who use ENDS predominantly. On each figure, the horizontal axis has the different levels of represented features while the vertical axis represents preferences. For each class, a higher number on the Y axis indicates a higher preference for the corresponding ENDS feature level on the X-axis

Relative model fit for the -2LL, Akaike Information Criterion (AIC), and BIC substantially improved between the 2 class (−2LL = − 30,254.06, AIC = 60,634.13, and BIC = 61,143.98) and 3 class (−2LL = − 29,578.59, AIC = 59,347.21, and BIC = 60,116.02) models [BIC χ^2^ difference (1) =1097.26, *p* < .001]. Model fit similarly improved between the 3 and 4 class (−2LL = 58,612.22, AIC = 58,612.22, and BIC = 59,640.00) models [BIC χ^2^ difference (1) =476.02 *p* < .001]. However, improvements in model became increasingly attenuated in models with a higher number of classes. Based on these observations, as well as on the tobacco use and sociodemographic differences between the 4 and 5 factor model classes, we ultimately identified the 4-class model as the best fitting model (Fig. 1. 4 Class Solution). Figure 1a, b and c represents the 4 classes for the features based on their overall themes with Fig. 1a representing features related to cost of use, Fig. 1b representing features under health effects and Fig. 1c for features related to the device. Although an individual’s true class membership cannot be definitively known, it is possible to compute probability of class membership using posterior probabilities, which are derived from the latent class measurement model [25]. The average maximum membership probability for the 4-class solution was 0.95548, which indicates minimal classification error.

### Class characterizations

The four identified classes were labeled based on their tobacco use characteristics. Tobacco cessation was important to 3 of the 4 classes and did not help distinguish the classes. These are distinguished as follows:

CLASS 1 – People with high nicotine dependence (*n* = 169, 27%) preferred ENDS products with device design similar to tobacco cigarettes. This class had 60% male, majority White (92%) and non-Latino (94%) compared to other classes. These individuals also had low rates of ENDS use, high cigarette use, had high dependence, and had the lowest rates of successful quit attempts compared to other classes.

CLASS 2 - People with moderate tobacco use (*n* = 159, 25%) were more interested in ENDS with low nicotine levels but less interested in ENDS products design. Like Class 1, this class skewed male (60%), but was more racially diverse (77%, White; 9% African American; 5% Asian; 8% other racial group). This group had a low rate of ENDS use and participants were less likely to anticipate ENDS use in the future. Participants in this group had lower levels of nicotine dependence compared with participants in groups 1 and 3 and had higher rates of success with quitting.

CLASS 3 - People who use ENDS and combustible tobacco (*n* = 147, 23%) were the most price conscious of all classes and more interested in flavors compared to all other classes. This class was as interested in ENDS products with design similar to cigarettes as Class 1 but comparatively less concerned about the relative health harms and negative effects of ENDS on their appearance. Class 3 had a more even distribution of gender (48% male) but was similar to Class 1 in terms of race/ethnicity. Class 3 also had the high ENDS use, high cigarette use, high dependence, and the highest rates of dual ENDS and combustible use among all classes.

CLASS 4 – People who use ENDS predominantly (*n* = 161, 25%) were largely insensitive to ENDS product features. There were modest effects noted for price, similarity to cigarettes, health harms, effects on appearance, and flavor, but these were substantially muted compared to the other classes. Class 4 was the most female (63%), most diverse (71% White, 26% Latino/Hispanic, 14% Asian, 7% other racial group), and the youngest compared to all other classes. ENDS use was the highest in this class (75% past-30-day use) but other tobacco product use for this group was generally lower. Those in class 4 had a moderate level of nicotine dependence compared to other classes.

## Discussion

This is the first research to identify preferences for different features of ENDS products by tobacco product use and sociodemographic factors. ENDS features that increased appeal were device design similar to cigarettes, low nicotine content, lower price, improved flavor. Class 1 members tend to be people with heavy cigarette use, who have had more trouble quitting and see appeal in ENDS products that are similar to cigarettes. Class 2 members tend to use cigarettes in more moderate amounts with lower levels of dependence and prefer products with lower nicotine level. Class 3 group members had the highest levels of dual ENDS and combustible tobacco use but were more price conscious, more interested in flavors, and less concerned about health effects. Class 4 members had high ENDS use but lower tobacco use and were largely insensitive to product features.

Class 1 group members showed preference for ENDS products that are similar to tobacco cigarettes. Research shows that there are certain sensorimotor features of ENDS products that increase their appeal to those who use cigarettes including airway stimulation, movement of hand-to-mouth and aerosol exhalation [26], making it easy to switch between the two. Some studies show increase in smoking cessation rates among smokers after starting using ENDS, suggesting that ENDS may be used as nicotine replacement therapy [27–29]. While the preferred goal is complete tobacco cessation, ENDS may be a useful harm-reduction strategy for those who cannot or will not quit nicotine. To support smokers who may benefit from ENDS, regulators may choose to encourage products that most closely mimic cigarettes [30].

Class 2 members preferred ENDS products with less nicotine and were less concerned about device design. Studies show that the nicotine content of ENDS can vary widely (from 0 to 35 mg/ml) [31, 32] and that nicotine content labels are not always accurate [5, 33]. Low levels of nicotine in ENDS products has been associated with trying ENDS products because it decreases the perception of harm [34]. Some people who use ENDS who try to quit smoking appreciate the ability to choose their nicotine levels and taper down their nicotine [35, 36]. However, people who use ENDS may compensate by using the e-cigarettes more often or inhaling more deeply. Another problem is that even when cartridges of ENDS products have the same level of nicotine, they may deliver different levels to people at different times, further leading to inconsistencies with the levels of nicotine that people think they are receiving with each use [33, 37]. It remains to be seen whether tapering nicotine levels in ENDS is a useful approach for reducing nicotine dependence, or whether it merely facilitates ENDS compensation or dual use of ENDS and combustible tobacco [38]. If low levels of nicotine support cessation of combustible cigarettes, then regulators will want to encourage the continued availability of different nicotine levels, ensure consistent labelling of nicotine content, and enact quality control to ensure that the nicotine level on the container matches the actual nicotine content.

Class 3 was made up of people with high levels of the use of both ENDS and tobacco products and a high level of nicotine dependence. This class was the most price conscious of the 4 classes. Pricing of tobacco products is a one of the most powerful tools in affecting tobacco product use. One study showed that an increase in price by 10% results in a 12% drop in sales of disposable ENDS and 18% decrease in sales for reusable ENDS [39]. States have the authority to regulate ENDS products and can set the minimum price by raising or lowering taxes on these products [40]. Although the FDA is unable tax ENDS products, regulations that increase cost would affect sale prices [41]. Studies also show cross-product price elasticity i.e. the relative price of cigarettes and e-cigarettes can affect the use of each [42]. Hence, if states lower taxes on ENDS, this will reduce the cost and may shift some people who use both products from the more harmful combustible tobacco products to the lower risk ENDS products.

Members of class 3 were also interested in flavors. Flavors strengthen the appeal of ENDS products and have also been associated with rewarding and reinforcing the value of ENDS products, hence increasing the desire to use [43–46]. Similar to our findings, research shows that people who use both ENDS and tobacco cigarettes have a higher likelihood of using flavored ENDS products compared with those who use a single product [30, 47]. In one study, people who use both products self-reported intention to reduce or quit ENDS use and increase tobacco cigarette use if restrictions were placed on ENDS flavors [48]. The challenge for regulators is to limit use while not overly deterring smokers from using ENDS for cessation or harm-reduction [49].

Our final class [4], comprised of people who use ENDS predominantly, had no strong preference for the ENDS features we offered. While ENDS use is very high in this class, these people may be less attached to ENDS than other classes. This class was made up of a more diverse and younger group (mean age 33 versus 42–46 for the other groups). This group may be more sensitive to attributes that are harder to measure, which may represent a unique challenge for policy makers. Alternatively, these participants may be willing to pay any price for ENDS and their use may not be affected by any particular ENDS feature. Therefore, any FDA regulatory action on ENDS may not affect their use of these products.

### Limitations

This study was used on an online convenience sample of U.S. participants drawn from a national survey panel and may not be representative of all those who use ENDS. However, tobacco control studies using convenience samples have tended to replicate the same patterns of statistical significance as nationally representative survey (albeit with different point estimates) [50]. Second, the preferences collected in the LCA are self-report and subject to social desirability and other biases. This data was collected in 2016 and the marketplace for ENDS has changed significantly with new products and modification of previously existing products. Some currently available ENDS products may have other features not present in this study, and choice experiments are limited by the number of attributes used, hence study findings may not be generalizable. In addition, preferences for hypothetical products may also not fully match what happens in real life when presented with a new product—marketing, peer use, accessibility, and other factors likely play a role. The LCA model assumes that patterns observed are typical of underlying subpopulations, which may not always be exact.

## Conclusion

FDA has a wide array of regulatory powers over ENDS, and states and localities have additional policy tools and approaches at their disposal. These regulatory decisions, including those regulating the characteristics of ENDS, likely affect who chooses to use ENDS and for what purpose. The present study provides a starting point about what characteristics matter and to whom. Knowing the preferences of different groups of people who use ENDS can allow policymakers to craft regulations that reduce ENDS among people who should not use them and allow ENDS use for people who need them.

## Data Availability

The datasets used and/or analyzed for this study are available from the corresponding author on reasonable request.
